# Cord blood vitamin A and vitamin D levels in relation to physical growth in exclusively breastfed infants aged 0-6 months

**DOI:** 10.3389/fendo.2024.1394408

**Published:** 2024-07-26

**Authors:** Wei Zhao, Chao Li, Wen Zhi Shen, Kai Yun Li, Yi Xi Cai, Feng Li, Hong Fu, Bin Peng, Jie Chen, Ting Yu Li, Li Chen

**Affiliations:** ^1^ Growth, Development and Mental Health Center of Children and Adolescents, Children’s Hospital of Chongqing Medical University; Chongqing Key Laboratory of Child Neurodevelopment and Cognitive Disorders; National Clinical Research Center for Child Health and Disorders, Ministry of Education Key Laboratory of Child Development and Disorders, Chongqing, China; ^2^ Department of Child Health Care, People's Hospital of Chongqing Liangjiang New Area, Chongqing, China; ^3^ Department of Child Health Care, Wanzhou District Health Center for Women and Children, Chongqing, China; ^4^ Department of Pediatrics, Chongqing University Jiangjin Hospital, Chongqing, China; ^5^ College of Public Health, Chongqing Medical University, Chongqing, China

**Keywords:** umbilical cord blood, vitamin A, vitamin D, exclusive breastfeeding, infants, physical growth

## Abstract

**Background:**

Vitamins A and D are essential for the health of pregnant women and infants. Nevertheless, the relationship between umbilical cord blood vitamins A and D levels and the physical growth of exclusively breastfed infants remains uncertain.

**Objective:**

This cohort study aims to examine the relationship between cord blood vitamins A and D levels and the physical growth of exclusively breastfed infants aged 0–6 months.

**Methods:**

140 singleton mother–infant pairs were recruited in total. Questionnaires were used to collect maternal and infant information, and liquid chromatography was utilized to quantify the levels of vitamins A and D in the umbilical cord blood. Anthropometric measurements were conducted at birth, at 3 and 6 months of age, and the weight-for-age z-score (WAZ), length-for-age z-score (LAZ), head circumference-for-age z-score (HAZ), and BMI-for-age z-score (BMIZ) were calculated. Univariate and multivariate linear regression models were used for the analysis.

**Results:**

The average concentration of vitamins A and D in cord blood was 0.58 ± 0.20 μmol/L and 34.07 ± 13.35 nmol/L, both below the normal range for children. After adjusting for confounding factors, vitamin A levels in cord blood positively correlated with HAZ growth in infants aged 3–6 months (β= 0.75, *P* < 0.01) while vitamin D levels negatively correlated with LAZ growth (β= −0.01, *P* = 0.01) and positively correlated with BMIZ growth (β= 0.02, *P* < 0.01).

**Conclusion:**

Higher Vitamin A levels at birth promote HAZ growth in infants aged 3–6 months while higher vitamin D levels at birth promote BMIZ growth in infants aged 3–6 months.

**Clinical trial registration:**

https://register.clinicaltrials.gov, identifier NCT04017286.

## 1 Introduction

Vitamin A insufficiency during pregnancy continues to pose a substantial public health concern in numerous developing nations. In 2014, the Chinese Nutrition Society recommended supplementation with vitamin A preparations in the mid to late stages of pregnancy to alleviate the health problems caused by vitamin A deficiency in pregnant women and fetuses (1). Despite these recommendations, a large-scale survey in China in 2022 found inadequate and deficient rates of 8.36%–10.38% for vitamin A during pregnancy, which was associated with insufficient intake of vitamin A-rich foods during pregnancy, improper use of vitamin A supplements, and inappropriate cooking methods (2). Vitamin A is crucial to visual development, organ and skeletal development, and maintenance of immune system function in fetuses and infants (3). Multiple studies have explored the association between cord blood vitamin A levels and anthropometric measurements at birth; however, the conclusions have been inconsistent. Ghebremeskel reported a significant and positive correlation between cord blood vitamin A levels and birth weight, head circumference, and length (4). But, another report mentioned that the cord blood vitamin A levels only positively correlated with birth weight, but not with head circumference or length at birth (5). Studies remain limited on the association between cord blood vitamin A levels and early infant growth.

Vitamin D deficiency during pregnancy is prevalent worldwide, with higher prevalence rates in Middle Eastern and Asian countries (6). In certain regions of China, during pregnancy, vitamin D insufficiency can affect up to 75% (7), and is primarily associated with factors such as the duration of sunlight exposure, dietary habits, skin pigmentation, and vitamin D supplementation (8). As we know, during gestational period, vitamin D is involved in the development of the fetal reproductive system, differentiation of adipocytes, and regulation of bone metabolism (9, 10), thus influencing the birth weight and skeletal development of the fetus (11). Increasing evidence suggests that vitamin D levels at birth affect the postnatal growth of offspring. Dalgard et al. observed that newborns with cord blood vitamin D concentrations >50 nmol/L were 0.49 cm longer (95% confidence interval [CI]: 0.05–0.93) than those with concentrations <12 nmol/L (12). However, a new finding pointed out that the levels of vitamin D in cord blood (>50 nmol/L) were linked to slower growth in infant length aged 0-6 months, with a decrease of 0.03 standard deviation scores in length for every 10 nmol/L increase in cord blood vitamin D concentration (95% CI:−0.05– −0.01) (13). Conversely, one report indicated that there was no association between cord blood vitamin D levels and anthropometric measurements of WAZ, LAZ, and BMIZ in infants (14). Similar findings were presented by Streym, who found cord blood vitamin D levels weren’t associated with WAZ growth during infancy (15). However, the latter two studies did not consider the influence of feeding practices (exclusive breastfeeding, mixed feeding, and formula feeding) or complementary foods.

Vitamin A and vitamin D are essential for maintaining the health of pregnant women and promoting the growth and development of fetuses and infants. Currently, research is lacking on the relationship between the cord blood levels of vitamins A and D and the physical growth of exclusively breastfed infants within the first six months. Therefore, our objective was to establish a prospective birth cohort to elucidate the levels of cord blood vitamins A and D and clarify the relationship between these levels and the physical growth of exclusively breastfed infants aged 0–6 months. The study results may provide effective clinical and theoretical references for maternal prenatal health and early life supplementation with vitamins A and D in infants.

## 2 Materials and methods

### 2.1 Study population

Healthy pregnant women attending obstetric clinics at Chongqing Wanzhou Health Center for Women and Children, People’s Hospital of Chongqing Liangjiang New Area, and Chongqing University Jiangjin Hospital in Chongqing, China, were recruited for this study from September 2018 to July 2021. After delivery, mother–infant pairs meeting the inclusion and exclusion criteria were enrolled in the study, with parents signing the informed consent form (parental version and infant version). Additionally, parents completed baseline characteristic questionnaires, including maternal age, prepregnancy height, prepregnancy weight, antenatal weight, smoking habits during pregnancy, folic acid supplementation during pregnancy, pregnancy complications, oral glucose tolerance test (OGTT) results, maternal education level, ethnicity, maternal occupation, pregnancy number, number of births, paternal height, and paternal weight. The infant baseline questionnaire included information on delivery mode, birth weight, birth date, sex, and gestational age at birth. All infants underwent follow-up visits at 3 and 6 months, during which anthropometric measurements (recumbent length, weight, and head circumference), vitamin supplementation (vitamins A and D) from birth to 6 months, and feeding practices were recorded.

### 2.2 Inclusion and exclusion criteria

The inclusion criteria were: pregnant women without malnutrition or chronic diseases, no history of long-term medication use, no family history of developmental disorders such as autism spectrum disorders, pregnant women without severe pregnancy complications, and pregnant women without neurological or psychiatric disorders, and singleton pregnancies. The exclusion criteria were: newborns with a history of perinatal asphyxia and a medical history severely affecting growth and development, and infants who were not regularly followed up. The withdrawal or termination criteria were miscarriage/induced abortion, or voluntary withdrawal.

The study has been approved by the Ethics Committee of the Children’s Hospital of Chongqing Medical University (Approval Number (2017): Institutional Review Board (STUDY) No. 43-1), of Chongqing Wanzhou Health Center for Women and Children [Approval Number: (2018-010)], of People ‘s Hospital of Chongqing Liangjiang New Area [Approval Number: (2018) Institutional Review Board (STUDY) No. 1], of Chongqing University Jiangjin Hospital [Approval Number: (20180913-1)].

### 2.3 Vitamins A and D measurement

During delivery, umbilical cord blood (1 mL) was immediately collected from each neonate. The blood samples were stored in the dark at 0°C–4°C. Samples were initially processed by centrifugation to remove proteins and impurities. Subsequently, an extracting agent was used to extract the target vitamins, and the resulting supernatant was redissolved using a mobile phase (methanol and pure water). The concentration of retinol (vitamin A) was determined at a flow rate of 1.0 mL/min using high-performance liquid chromatography (Agilent, USA). The concentration of 25(OH)D [sum of 25(OH)D2 and D3] was measured at a flow rate of 0.6 mL/min using liquid chromatography-tandem mass spectrometry (LC–MS/MS 8040; Shimadzu, Japan). Standard curve equations were established based on the measured concentrations of standard substances. The results for the quality control (QC) and test samples were calculated using these equations. A batch is considered acceptable if the QC values fall within the range of X ± 3D, and the test results for the samples in that batch are reported.

### 2.4 Anthropometric measurement at birth and during infancy

In a dedicated room, measurements were conducted by pediatric specialty nurses who had undergone standardized training and demonstrated competence through assessments. For the weight and recumbent length measurements, infants were dressed only in underwear without shoes or diapers. Measured values were adjusted by subtracting the underwear weight to obtain the final weight of the accepted infants. Precision measurements were performed using precise infant examination instruments with an accuracy of 0.01 kg and 1 mm. Head circumference was measured using a nonstretchable tape along the upper edge of the infant’s right eyebrow, passing over the right ear above the occipital prominence, and returning to the zero point at the intersection of the left eyebrow with an accuracy of 1 mm. All measurements were performed three consecutive times, and the average value was used. We used the World Health Organization software 2011 V.3.2.2 for WAZ, LAZ, HAZ and BMIZ at each of the three measurement time points.

### 2.5 Vitamin A and D supplementation

All gravida were instructed to regularly take vitamin D3 at a dose of 400 IU/day upon enrollment, with a recommendation for oral intake of various vitamin formulations containing vitamin A. To follow the infant nutrition and health guidelines (16), all infants commenced vitamin D3 supplementation within the first week after birth, with a minimum daily intake of 400 IU. In this study, infant vitamin D supplementation was categorized into three methods: 90.0% of infants received oral vitamin AD combination drops (daily intake of VA 450 µg RAE/d and VD3 500 IU); 6.4% of infants alternated between oral vitamin AD combination drops (daily intake of VA 450 µg RAE/d and VD3 500 IU) and vitamin D3 drops (daily intake of VD3 400 IU); and 2.9% of infants received oral vitamin D3 drops (daily intake of VD3 400 IU).

### 2.6 Variables and definitions

The formula for calculating body mass index (BMI) was weight (kg) divided by height (m) squared (kg/m²). Similarly, the formula for calculating gestational weight increase was weight at delivery minus pre-pregnancy weight (kg). Infant indices of 3–6 months [WAZ(3–6), LAZ(3–6), HAZ(3–6), and BMIZ(3–6)] were calculated by subtracting the Z scores at 3 months from those at 6 months, whereas indices of 0–3 months [WAZ(0–3)] were determined by subtracting Z scores at birth from those at 3 months.

### 2.7 Statistical analysis

Categorical variables are reported as frequencies (percentages), and continuous variables are shown as mean ± standard deviation (SD). Univariate and multivariate linear regression models were used to explore the association between cord blood vitamin A and vitamin D levels and infant physical growth. Furthermore, we examined the interaction between cord blood vitamins A and D. Outcome variables included WAZ(0–3), WAZ(3–6), LAZ(3–6), HAZ(3–6), and BMIZ(3–6) within the first six months of infancy. Confounding factors were determined based on a literature review and professional knowledge.

Two linear regression models were used. The tables show the unadjusted Model 1 and adjusted Model 2. Covariates corrected in WAZ/HAZ/BMIZ multivariate linear regression models encompassed maternal age, prepregnancy BMI, gestational weight gain, gestational age, birth weight, pregnancy complications (present or absent), fetal sex (male or female), and birth season (spring, summer, autumn, or winter), vitamin A and vitamin D supplement (VA 450 µg RAE/d and VD3 500 IU/d, alternated between (VA 450 µg RAE/d and VD3 500 IU/d) and (VD3 400 IU/d), VD3 400 IU/d). Covariates adjusted in the LAZ multivariate linear regression model included maternal age, maternal height, paternal height, gestational weight gain, gestational age, birth weight, pregnancy complications (present or absent), fetal sex (male or female), and season of birth (spring, summer, autumn, and winter), vitamin A and vitamin D supplement [VA 450 µg RAE/d and VD3 500 IU/d, alternated between (VA 450 µg RAE/d and VD3 500 IU/d) and (VD3 400 IU/d), VD3 400 IU/d]. Residuals were plotted using linear regression models to assess the normality and homoscedasticity. Statistical analysis were described using regression coefficients (β estimates) and corresponding 95% CI. IBM SPSS Statistics for Windows version 27 was applied for all analyses. *P* < 0.05 was significance.

## 3 Results

### 3.1 Baseline characteristics of the mother–infant pairs

**Figure 1:**
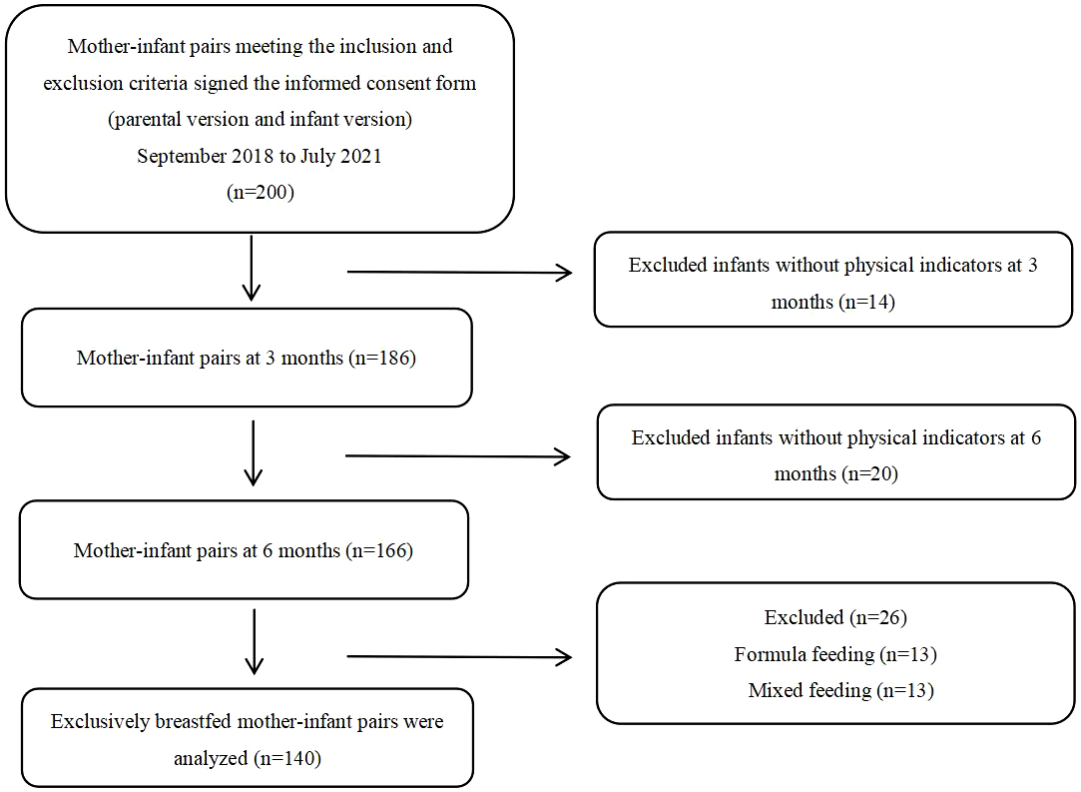
Participant flowchart.

This prospective cohort study initially enrolled 200 mother–infant pairs. However, during the study, 60 pairs were excluded for reasons such as inability to obtain infant anthropometric measurements at 3 months (n = 14), inability to obtain infant anthropometric measurements at 6 months (n = 20), formula feeding (n = 13), and mixed feeding (n = 13). Consequently, 140 mother–infant pairs were examined. **Figure 1** displayed the flowchart of participants.

The descriptive characteristics of the parents and infants in this cohort are presented in **Tables 1**, **2**. The average concentration of vitamin A was 0.58 ± 0.20 μmol/L, which was below the normal level for children (≥1.05 μmol/L). The average concentration of vitamin D was 34.07 ± 13.35 nmol/L, falling below the standard for sufficient vitamin D levels in children (>50 nmol/L). During the follow-up period, the supplementation rates of infant vitamin A and vitamin D3 were 96.45% and 99.29%, respectively.

**Table 1:** Parental demographic characteristics.

		N	(%)
Maternal age	<35	123	87.9
	≥35	17	12.1
Parity	≤1	47	33.6
	>1	93	66.4
Gravidity	primipara	80	57.1
	multipara	60	42.9
Pregnancy complications	no complications	100	71.4
	GDM^b^	20	14.3
	GDM combined with obesity	3	2.1
	hypothyroidism	5	3.6
	hypothyroidism combined with obesity	3	2.1
	gestational hypertension	4	2.9
	other	5	3.6
Pre-pregnancy BMI^a^	underweight	21	15.0
	normal	96	68.6
	overweight	23	16.4
Gestational weight gain	inadequate	43	30.7
	appropriate	73	52.2
	excessive	24	17.1
Paternal BMI	underweight	2	1.4
	normal	74	52.9
	overweight	64	45.7

Values are mean ± SD, or n (%).

a BMI, body mass index.

b GDM, gestational diabetes mellitus.

**Table 2:** Infant demographic characteristics.

		N	(%)
Birth weight	low birth weight	5	3.6
	normal birth weight	128	91.4
	macrosomia	7	5.0
Birth season	spring	6	4.3
	summer	35	25.0
	autumn	86	61.4
	winter	13	9.3
Gender	male	68	48.6
	female	72	51.4
Gestational	preterm birth	3	2.1
	full-term birth	137	97.9
Delivery mode	vaginal	86	61.4
	cesarean section	54	38.6
Vitamin and vitamin D supplement	no supplement	1	0.7
	VA 450 µg RAE/d^a^ and VD3 500 IU/d	126	90.0
	alternated between (VA 450 µg RAE/d and VD3 500 IU/d) and (VD3 400 IU/d)	9	6.4
	VD3 400 IU/d	4	2.9

Values are mean ± SD, or n (%).

a RAE, retinol activity equivalents.

### 3.2 Umbilical cord blood vitamin A and infant anthropometric growth

Multivariate linear regression analysis revealed a positive correlation between cord blood vitamin A and HAZ growth in infants aged 3–6 months (β = 0.75, *P* < 0.01). No significant correlation was present between vitamin A and WAZ growth in infants aged 0–3 months or between WAZ, LAZ, and BMIZ growth in infants aged 3–6 months (**Table 3**). Umbilical cord blood vitamin A levels were categorized according to WHO and “Child Health Science” (4th edition) (17, 18): The percentage of infants with umbilical cord blood vitamin A of 0.70 μmol/L-1.05 μmol/L was 23.57%, 0.35 μmol/L-0.70 μmol/L was 68.57%, and <0.35 μmol/L was 6.42%.

**Table 3:** Umbilical cord blood vitamin A and infant anthropometric growth.

	VA^a,b^			VA^a,c^		
	β	(95% CI)	*P*	β	(95% CI)	*P*
WAZ (0-3)	-0.55	(-1.32 - 0.23)	0.16	-0.39	(-1.17 - 0.39)	0.33
WAZ (3-6)	0.06	(-0.44 - 0.56)	0.82	0.24	(-0.31 - 0.78)	0.39
LAZ (3-6)	0.55	(-0.05 - 1.14)	0.07	0.67	(-0.01 - 1.34)	0.05
HAZ (3-6)	0.75	(0.29 - 1.20)	<0.01	0.75	(0.24 - 1.27)	<0.01
BMIZ (3-6)	-0.21	(-0.89 - 0.47)	0.54	-0.03	(-0.79 - 0.73)	0.93

Data are presented as β and 95%CI.

a VA, umbilical cord blood vitamin A.

WAZ, weight-for-age z-scores; LAZ, length-for-age z-scores; HAZ, head circumference-for-age z-score; BMIZ, BMI-for-age z-scores.

b Model 1: unadjusted.

c Model 2: adjusted for maternal age, pre-pregnancy BMI, maternal height, paternal height, gestational weight gain, gestational age, birth weight, pregnancy complications (present, absent), fetal sex (male, female), and season of birth (spring, summer, autumn, winter), vitamin A and vitamin D supplement (VA 450 µg RAE/d and VD3 500 IU/d, alternated between (VA 450 µg RAE/d and VD3 500 IU/d) and (VD3 400 IU/d), VD3 400 IU/d).

Statistical significance was set at P < 0.05.

### 3.3 Umbilical cord blood vitamin D and infant anthropometric growth

Multivariate linear regression analysis demonstrated a negative correlation between cord blood vitamin D and LAZ growth in infants aged 3–6 months (β = −0.01, *P* = 0.01), a positive correlation between vitamin D and BMIZ growth in infants aged 3–6 months (β = 0.02, *P* < 0.01). No significant correlation was observed between vitamin D and WAZ growth in infants aged 0–3 months, or between WAZ and HAZ growth in infants aged 3–6 months (**Table 4**). Umbilical cord blood vitamin D levels were categorized based on WHO and “Endocrine Society clinical practice guideline” (19, 20): The percentage of infants with umbilical cord blood vitamin D in infants was <50 nmol/L in 87.86%, 50 nmol/L-72.5 nmol/L in 11.43%.

**Table 4:** Umbilical cord blood vitamin D and infant anthropometric growth.

	VD^a,b^			VD^a,c^		
	β	(95% CI)	*P*	β	(95% CI)	*P*
WAZ (0-3)	0.00	(-0.01 - 0.01)	0.84	0.00	(-0.02 - 0.01)	0.46
WAZ (3-6)	0.01	(0.00 - 0.01)	0.13	0.01	(0.00 - 0.01)	0.29
LAZ (3-6)	-0.01	(-0.02 - 0.00)	0.06	-0.01	(-0.02 - 0.00)	0.01
HAZ (3-6)	0.00	(0.00 - 0.01)	0.36	0.00	(-0.01 - 0.01)	0.55
BMIZ (3-6)	0.02	(0.01 - 0.03)	<0.01	0.02	(0.01 - 0.03)	<0.01

Data are presented as β and 95%CI.

a VD, umbilical cord blood vitamin D.

WAZ, weight-for-age z-scores; LAZ, length-for-age z-scores; HAZ, head circumference-for-age z-score; BMIZ, BMI-for-age z-scores.

b Model 1: unadjusted.

c Model 2: adjusted for maternal age, pre-pregnancy BMI, maternal height, paternal height, gestational weight gain, gestational age, birth weight, pregnancy complications (present, absent), fetal sex (male, female), and season of birth (spring, summer, autumn, winter), vitamin A and vitamin D supplement (VA 450 µg RAE/d and VD3 500 IU/d, alternated between (VA 450 µg RAE/d and VD3 500 IU/d) and (VD3 400 IU/d), VD3 400 IU/d).

Statistical significance was set at P < 0.05.

## 4 Discussion

This cohort finding investigated the association between cord blood levels of vitamins A and D and the physical growth of exclusively breastfed infants aged 0–3 and 3–6 months of age. We discovered that the average concentrations of vitamin A and vitamin D in cord blood were both below the normal values for children. Higher vitamin A levels were linked to more noticeable HAZ growth in infants aged 3–6 months. Additionally, lower vitamin D levels had been related to increased LAZ growth and lower BMIZ growth in infants aged 3–6 months. Importantly, these findings were independent of maternal prepregnancy BMI and gestational weight gain.

In our study, the mean of cord blood vitamin A was 0.58 ± 0.20 μmol/L, with 98.56% of newborns having vitamin A levels <1.05 μmol/L. This may be related to the placental transport mechanism of vitamin A and the maternal intake of vitamin A during pregnancy. Owing to the absence of strict requirements for pregnant women to take various daily vitamin formulations containing vitamin A, intermittent use of such products may occur. Additionally, as vitamin A is a fat-soluble vitamin with a lower placental transfer rate, cord blood vitamin A levels are generally lower than maternal levels. The geographical location and latitude of India are similar to those of Chongqing, where vitamin D levels were generally low at birth, with at least 95.7% of newborns having vitamin D levels <50 nmol/L (21). Similarly, in our study, the average concentration of umbilical cord blood vitamin D was 34.07 ± 13.35 nmol/L, which was lower than 50 nmol/L, and the neonates with vitamin D deficiency in umbilical cord blood were still as high as 87.86%. This could be linked to the truth that approximately half of the vitamin D transported through the placenta is derived from the maternal peripheral blood. Furthermore, the unique topography and mountainous climate of the Chongqing region, characterized by reduced sunlight duration and intensity, may also be a contributing factor to the diminished levels of cord blood vitamin D. Our study findings indicate that the prevalence of vitamin D deficiency remains relatively high among pregnant women and their offspring at birth in our region. Appropriate vitamin D supplementation is important to maintain the optimal vitamin D status of pregnant women. Combined with the requirements of international guidelines and Chinese guidelines (19, 22), we recommend that pregnant women in our country supplement with a minimum of 600 IU of vitamin D daily to enhance umbilical cord blood vitamin D levels (23), thereby addressing neonatal vitamin D insufficiency and deficiency.

Our results revealed a positive correlation between cord blood vitamin A levels and HAZ growth in exclusively breastfed infants aged 3–6 months. Specifically, for each 1 μmol/L increase in cord blood vitamin A concentration, infants between 3–6 months of age experienced a 0.75-unit growth in HAZ. Previous research had investigated the connection between cord blood vitamin A levels and head circumference at birth. In 1994, Ghebremeskel observed a relationship between vitamin A levels in cord blood and head circumference at birth(r = 0.322, *P* = 0.004) (4). Subsequently, a prospective cohort study by Elizabeth et al. revealed a substantial positive correlation between umbilical cord blood retinol levels and birth head circumference (β = 0.01, *P* = 0.03) (24). However, several studies have suggested a lack of correlation (5). Our results indicate that higher cord blood vitamin A levels are beneficial for the growth rate of infant head circumference, although the underlying mechanisms remain unclear. Vitamin A promotes cranial bone development, which is linked to its involvement in bone absorption and reconstruction (25–27). Previous animal studies have suggested that both excessively high and deficient serum vitamin A levels in mice can impede cranial bone development (28–30), emphasizing the importance of optimal vitamin A concentrations in facilitating cranial bone development, and that as cord blood vitamin A is transported from the placenta, its levels correlate with maternal vitamin A levels during pregnancy (31). Research indicates that adequate vitamin A intake in the late stages of pregnancy contributes to elevated cord blood vitamin A levels in newborns (5), promoting fetal and neonatal growth (32). Vitamin A deficiency is more likely to occur due to the accelerated growth of the fetus in late pregnancy; therefore, the World Health Organization recommends initiating low-dose vitamin A supplementation from mid to late pregnancy (3). We propose that appropriate supplementation of vitamin A during pregnancy to maintain higher cord blood vitamin A levels is advantageous for the early growth of infant head circumference.

Additionally, this research showed a negative relationship between LAZ growth in infants aged 3–6 months and vitamin D levels. Specifically, for each 1 nmol/L reduction in cord blood vitamin D concentration, an additional 0.01 increase occurred in LAZ in infants aged 3–6 months. The expert consensus on the clinical application of vitamins A and D in Chinese children recommended vitamin D supplementation for infants starting from the first week of life (800 IU for preterm infants and 400 IU for full-term newborns) (33), aiming to promote the growth of infants with vitamin D deficiency (34). Our findings indicated that, under conditions of generally low cord blood vitamin D levels, infants commence supplementation with the recommended dose of vitamin D within 1–2 weeks after birth (supplementation rate of 99.29%), and that those with lower vitamin D levels at birth exhibited a more pronounced increase in LAZ aged 3–6 months. This aligns with the outcomes of previous research. In India, where vitamin D insufficiency is prevalent, one finding emphasized that weekly supplementation with 1400 IU of vitamin D3 can improve linear growth in low-birth-weight infants (35). However, Wang believed that there was no correlation between LAZ in infants and cord blood vitamin D levels, which may be attributed to variations in the grouping criteria for cord blood vitamin D levels and the lack of adjustments for confounding factors, such as feeding practices and complementary food (14). A prospective multi-ethnic cohort finding showed that offspring of mothers with prenatal vitamin D deficiency exhibited accelerated height growth during infancy, which was considered to indicate a link with postnatal vitamin D supplementation in infants; nevertheless, the details of infant vitamin D supplementation were not examined in this study (36). Interestingly, Hauta–Alus team revealed that slower infant growth aged 0–6 months was linked to the cord blood vitamin D levels (>50 nmol/L), and an inverted U-shaped association could exist between vitamin D levels and infant growth; both excessively high and low vitamin D levels were associated with hindrances in infant physical development (13). Therefore, our team hypothesized that infants with lower cord blood vitamin D levels may experience a greater impact from early postnatal vitamin D supplementation than those with higher levels, promoting accelerated bone development and height increase. The mechanism may be related to the kinetics of vitamin D. The lower the baseline serum vitamin D_3_ level, the more pronounced the increase in 25(OH)D levels after vitamin D_3_ supplementation (37). Although postnatal vitamin D supplementation in infants can compensate for maternal vitamin D deficiency during pregnancy, it is crucial to acknowledge the close association between prenatal vitamin D deficiency and adverse outcomes in fetal and neonatal development (6). Hence, we recommend that gravidas, not only increasing their outdoor activities and exposure to sunlight, but also considering supplementation with an adequate amount of vitamin D to preserve normal serum vitamin D levels and foster early growth and development in infants.

Besides, BMIZ growth aged 3–6 months was favorably linked with vitamin D levels. Thus, BMIZ growth decreases by 0.02 for every 1 nmol/L decrease in cord blood vitamin D concentration in infants aged 3–6 months. The past studies didn’t find a correlation between cord blood vitamin D levels and WAZ, BMIZ, or body composition (fat and fat-free mass) at 5 months; however, these studies did not adjust for feeding practices (14, 38). Weiler revealed that infants with lower cord blood vitamin D levels weighed more at birth and within the first 15 days postnatally than infants with higher levels (39). Conversely, infants with fewer levels of vitamin D at birth exhibited slower BMIZ growth between 3 and 6 months of age. The primary source of vitamin D in the human body is synthesis in the skin through exposure to sunlight (UV radiation), followed by vitamin D supplement and dietary intake, although few foods contain significant amounts of vitamin D, moreover, breast milk contains minim amounts of vitamin D (8). One finding conducted in Indonesia revealed that 87% of offspring with vitamin D deficiency (<50 nmol/L) at birth experienced a rise in serum vitamin D levels to normal (≥50 nmol/L) at the age of 6 months after prolonged exposure to sunlight postnatally, whereas only 67% of offspring with sufficient cord blood vitamin D exhibited an increase in serum vitamin D levels. This phenomenon indicates that serum vitamin D levels rise more in infants with cord blood vitamin D deficiency from 0 to 6 months of age (40). Another systematic review and meta-analysis on infant vitamin D supplementation indicated that infants with baseline 25(OH)D concentrations <50 nmol/L experienced a more noteworthy increase in serum 25(OH)D concentrations after supplementation with vitamin D2/D3 (100–1600 IU/d) than infants with baseline 25(OH)D concentrations >50 nmol/L (58.2 nmol/L, 95%CI: 49.7–66.6, vs. 28.6 nmol/L, 95%CI: 16.4–40.8; *P* = 0.001) (41). These may be related to the kinetics of vitamin D (37). Vitamin D is stored in adipocytes, and adipose tissue regulates its slow release (42). Simultaneously, vitamin D inhibits adipocyte formation of adipocytes (10). Therefore, we speculated that infants with lower vitamin D levels at birth might exhibit a more pronounced rise in serum vitamin D levels and have with a potentially more prominent inhibitory effect on adipocyte differentiation and formation, leading to decelerated BMIZ growth. A systematic review revealed a significant reduction in the BMIZ at 3–6 years of age in offspring supplemented with vitamin D during pregnancy or infancy compared with BMIZ in offspring in the placebo or control groups (43). In our investigation, infants with lower vitamin D levels at birth may have experienced a substantial elevate in serum vitamin D levels after postnatal supplementation. However, whether the BMIZ growth in infants continues to slow with age requires further clarification.

This study has clinical significance. No interaction was observed between cord blood vitamin A and cord blood vitamin D levels, confirming the independent impact of these factors on infant physical growth outcomes. Furthermore, while existing research has explored the relationship between cord blood vitamins A and D levels and offspring physical growth, prospective cohort studies examining the connection between these levels and early-life physical growth in exclusively breastfed infants are rare. For the analysis of the dependent and independent variables in this study, we used continuous variables rather than categorical variables, thereby maximizing data integrity and avoiding information loss due to discretization. Importantly, our primary focus was on the physical growth trajectory between the two time points during the rapid growth phase of infants as opposed to assessing physical indicators at fixed time points, an aspect seldom addressed in other studies.

There were certain restrictions on this investigation. First, the length and head circumference of the offspring at birth were measured by obstetric nurses with nonuniform training while subsequent follow-ups were conducted by pediatric nurses with standardized training. To ensure accuracy, the data on birth length and head circumference were not included in the statistical analysis. Second, the sample size in this study was relatively small, and the duration was short. Subsequent research should involve larger sample sizes, multicenter collaborations, and longer follow-up periods to validate the effect of cord blood vitamins A and D on infant physical growth. Third, a potential for significant recall bias exists because of the extended duration of maternal pregnancy and the complexity of oral supplementation with vitamin A and vitamin D, so we could not obtain detailed and accurate supplementation information. Furthermore, our study encompassed infants aged 0-6 months, and due to parental reluctance to subject their healthy infants to frequent blood draws within a short timeframe, as well as ethical considerations, we were unable to obtain vitamins A and D levels from infants at 3 and 6 months of age. Additionally, due to the postpartum confinement practices in China, we were unable to fully investigate the dietary status of postpartum mothers. Despite the minimal presence of vitamin D in breast milk, this may also be a contributing factor. Lastly, although most infants in this cohort were born in the fall and winter seasons (70.9%), we corrected for the season of birth as a confounding factor; however, we did not collect data on the duration of sunlight exposure for the infants.

In summary, the levels of vitamin A at birth promote HAZ growth in infants aged 3–6 months while vitamin D levels facilitate BMIZ growth during the same period but were negatively correlated with LAZ. This discovery contributes to raising awareness among healthcare professionals and parents regarding the nutritional intake and monitoring of vitamins A and D during pregnancy. It elucidates the association between umbilical cord blood levels of vitamins A and D and infant physical growth, and assists clinical staff in better explaining the differences in infant physical growth to parents. We recommend adequate maternal nutrition during pregnancy, increased exposure to sunlight, and moderate supplementation of vitamins A and D are beneficial for infant growth. Continued follow-up is necessary in later stages to assess the extended growth trajectory of the infant and to provide data to support maternal health management during pregnancy.

## Data availability statement

The raw data supporting the conclusions of this article will be made available by the authors, without undue reservation.

## Ethics statement

The study has been approved by the Ethics Committee of the Children’s Hospital of Chongqing Medical University [Approval Number: (2017) Institutional Review Board (STUDY) No. 43-1], of Chongqing Wanzhou Health Center for Women and Children [Approval Number: (2018-010)], of People ‘s Hospital of Chongqing Liangjiang New Area [Approval Number: (2018) Institutional Review Board (STUDY) No. 1], of Chongqing University Jiangjin Hospital (Approval Number: (20180913-1)). The studies were conducted in accordance with the local legislation and institutional requirements. Written informed consent for participation in this study was provided by the participants’ legal guardians/next of kin.

## Author contributions

WZ: Writing – original draft, Data curation, Formal analysis, Investigation, Methodology. CL: Writing – review & editing, Data curation, Investigation, Supervision. WS: Project administration, Supervision, Writing – original draft, Data curation. KL: Investigation, Writing – original draft, Project administration. YC: Project administration, Supervision, Writing – original draft, Data curation. FL: Project administration, Supervision, Writing – original draft. HF: Data curation, Investigation, Writing – original draft. BP: Data curation, Formal analysis, Writing – review & editing. JC: Project administration, Writing – review & editing, Validation. TL: Project administration, Resources, Writing – review & editing. LC: Writing – review & editing, Conceptualization, Funding acquisition, Methodology, Project administration.

## References

[1] CNS. Chinese residents dietary nutrient reference intake quick check manual. Beijing: China Standards Press (2014).

[2] NHC. Vitamin a and vitamine levels in chinese pregnant women and gestational diseases. Beijing: People 's Health Press (2022).

[3] McGuire S. WHO Guideline: Vitamin A Supplementation in Pregnant Women. Geneva: World Health Organization. Geneva: World Health Organization. Adv Nutr. (2011) 3:215–6. doi: 10.3945/an.111.001701 PMC3648723 22516730

[4] Ghebremeskel K Burns L Burden TJ Harbige L Costeloe K Powell JJ. Vitamin a and related essential nutrients in cord blood: relationships with anthropometric measurements at birth. Early Hum Dev. (1994) 39:177–88. doi: 10.1016/0378-3782(94)90196-1 7712952

[5] Huan L Jingkun M Jie C Tingyu L. Effects of dietary vitamin a intake in late pregnancy on the neonatal vitamin a level and related factors. J Bio-education. (2018) 6. doi: 10.3969/j.issn.2095-4301.2018.03.004

[6] WHO. WHO antenatal care recommendations for a positive pregnancy experience: Nutritional interventions update: Vitamin D supplements during pregnancy [Internet]. Geneva: World Health Organization (2020). 32783436

[7] Dong J Zhou Q Wang J Lu Y Li J Wang L. Association between variants in vitamin D-binding protein gene and vitamin D deficiency among pregnant women in China. J Clin Lab Anal. (2020) 34:e23376. doi: 10.1002/jcla.23376 32537819 PMC7521226

[8] Mansur JL Oliveri B Giacoia E Fusaro D Costanzo PR. Vitamin D: before, during and after pregnancy: effect on neonates and children. Nutrients. (2022) 14:1900. doi: 10.3390/nu14091900 35565867 PMC9105305

[9] Lerchbaum E Obermayer-Pietsch B. Vitamin D and fertility: A systematic review. Eur J Endocrinol. (2012) 166:765–78. doi: 10.1530/EJE-11-0984 22275473

[10] Wood RJ. Vitamin D. and adipogenesis: new molecular insights. Nutr Rev. (2008) 66:40–6. doi: 10.1111/j.1753-4887.2007.00004.x 18254883

[11] Luo LM Wu N Zhang J Yang D. Maternal vitamin D levels correlate with fetal weight and bone metabolism during pregnancy: A materno-neonatal analysis of bone metabolism parameters. J Perinat Med. (2023) 51:538–45. doi: 10.1515/jpm-2022-0068 36435526

[12] Dalgård C Petersen MS Steuerwald U Weihe P Grandjean P. Umbilical cord serum 25-hydroxyvitamin D concentrations and relation to birthweight, head circumference and infant length at age 14 days. Paediatr Perinat Epidemiol. (2016) 30:238–45. doi: 10.1111/ppe.12288 PMC6172952 27038010

[13] Hauta-Alus HH Kajantie E Holmlund-Suila EM Rosendahl J Valkama SM Enlund-Cerullo M. High pregnancy, cord blood, and infant vitamin D concentrations may predict slower infant growth. J Clin Endocrinol Metab. (2019) 104:397–407. doi: 10.1210/jc.2018-00602 30247704

[14] Wang H Yu XD Huang LS Chen Q Ouyang FX Wang X. Fetal vitamin D concentration and growth, adiposity and neurodevelopment during infancy. Eur J Clin Nutr. (2018) 72:1396–403. doi: 10.1038/s41430-017-0075-9 29348623

[15] Við Streym S Kristine Moller U Rejnmark L Heickendorff L Mosekilde L Vestergaard P. Maternal and infant vitamin D status during the first 9 months of infant life-a cohort study. Eur J Clin Nutr. (2013) 67:1022–8. doi: 10.1038/ejcn.2013.152 24002039

[16] CNS. Pagoda of balanced diet for women and children helps promote healthy China, nutrition first. Beijing: Chinese Nutrition Society (2018).

[17] Meng M Fan j Tingyu Li. Child health science. 4th Edition. Beijing: People 's Health Publishing House (2020).

[18] WHO. Vitamin A deficiency. Geneva: World Health Organization (2009).

[19] Holick MF Binkley NC Bischoff-Ferrari HA Gordon CM Hanley DA Heaney RP. Evaluation, treatment, and prevention of vitamin D deficiency: an endocrine society clinical practice guideline. J Clin Endocrinol Metab. (2011) 96:1911–30. doi: 10.1210/jc.2011-0385 21646368

[20] WHO. Vitamin D supplementation for infants. Geneva: World Health Organization (eLENA) (2017).

[21] Trilok Kumar G Chugh R Eggersdorfer M. Poor vitamin D status in healthy populations in India: A review of current evidence. Int J Vitam Nutr Res. (2015) 85:185–201. doi: 10.1024/0300-9831/a000228 26575971

[22] Płudowski P Kos-Kudła B Walczak M Fal A Zozulińska-Ziółkiewicz D Sieroszewski P. Guidelines for preventing and treating vitamin D deficiency: A 2023 update in Poland. Nutrients. (2023) 15:695. doi: 10.3390/nu15030695 36771403 PMC9920487

[23] Harreiter J Mendoza LC Simmons D Desoye G Devlieger R Galjaard S. Vitamin D3 supplementation in overweight/obese pregnant women: no effects on the maternal or fetal lipid profile and body fat distribution-a secondary analysis of the multicentric, randomized, controlled vitamin D and lifestyle for gestational diabetes prevention trial (Dali). Nutrients. (2022) 14:3781. doi: 10.3390/nu14183781 36145157 PMC9503968

[24] Masters ET Jedrychowski W Schleicher RL Tsai WY Tu YH Camann D. Relation between prenatal lipid-soluble micronutrient status, environmental pollutant exposure, and birth outcomes. Am J Clin Nutr. (2007) 86:1139–45. doi: 10.1093/ajcn/86.4.1139 PMC2082133 17921394

[25] Conaway HH Pirhayati A Persson E Pettersson U Svensson O Lindholm C. Retinoids stimulate periosteal bone resorption by enhancing the protein rankl, a response inhibited by monomeric glucocorticoid receptor. J Biol Chem. (2011) 286:31425–36. doi: 10.1074/jbc.M111.247734 PMC3173101 21715325

[26] Yorgan TA Heckt T Rendenbach C Helmis C Seitz S Streichert T. Immediate effects of retinoic acid on gene expression in primary murine osteoblasts. J Bone Miner Metab. (2016) 34:161–70. doi: 10.1007/s00774-015-0666-2 25956707

[27] Dingwall M Marchildon F Gunanayagam A Louis CS Wiper-Bergeron N. Retinoic acid-induced smad3 expression is required for the induction of osteoblastogenesis of mesenchymal stem cells. Differentiation. (2011) 82:57–65. doi: 10.1016/j.diff.2011.05.003 21612856

[28] Krutzen C Roa LA Bloemen M Von den Hoff JW. Excess vitamin a might contribute to submucous clefting by inhibiting wnt-mediated bone formation. Orthod Craniofac Res. (2023) 26:132–9. doi: 10.1111/ocr.12594 PMC10084165 35716278

[29] Kneissel M Studer A Cortesi R Susa M. Retinoid-induced bone thinning is caused by subperiosteal osteoclast activity in adult rodents. Bone. (2005) 36:202–14. doi: 10.1016/j.bone.2004.11.006 15780946

[30] Dai Q Sun S Jin A Gong X Xu H Yang Y. Osteoblastic rar inhibition causes vad-like craniofacial skeletal deformity. J Dent Res. (2023) 102:667–77. doi: 10.1177/00220345231151691 37036085

[31] Azaïs-Braesco V Pascal G. Vitamin a in pregnancy: requirements and safety limits. Am J Clin Nutr. (2000) 71:1325s–33s. doi: 10.1093/ajcn/71.5.1325s 10799410

[32] Ma G Chen Y Liu X Gao Y Deavila JM Zhu MJ. Vitamin a supplementation during pregnancy in shaping child growth outcomes: A meta-analysis. Crit Rev Food Sci Nutr. (2022) 63:12240–55. doi: 10.1080/10408398.2022.2099810 PMC9849478 35852163

[33] CPMA. Expert consensus on clinical application of vitamin a and vitamin D in chinese children. Chin J Child Health Care. (2021) 29:110–6. doi: 10.11852/zgetbjzz2020-2118

[34] Gat-Yablonski G Phillip M. Nutritionally-induced catch-up growth. Nutrients. (2015) 7:517–51. doi: 10.3390/nu7010517 PMC4303852 25594438

[35] Kumar GT Sachdev HS Chellani H Rehman AM Singh V Arora H. Effect of weekly vitamin D supplements on mortality, morbidity, and growth of low birthweight term infants in India up to age 6 months: randomised controlled trial. Bmj. (2011) 342:d2975. doi: 10.1136/bmj.d2975 21628364 PMC3104477

[36] Leffelaar ER Vrijkotte TG van Eijsden M. Maternal early pregnancy vitamin D status in relation to fetal and neonatal growth: results of the multi-ethnic amsterdam born children and their development cohort. Br J Nutr. (2010) 104:108–17. doi: 10.1017/S000711451000022X 20193097

[37] Heaney RP Armas LA Shary JR Bell NH Binkley N Hollis BW. 25-hydroxylation of vitamin D3: relation to circulating vitamin D3 under various input conditions. Am J Clin Nutr. (2008) 87:1738–42. doi: 10.1093/ajcn/87.6.1738 18541563

[38] Sauder KA Koeppen HJ Shapiro ALB Kalata KE Stamatoiu AV Ringham BM. Prenatal vitamin D intake, cord blood 25-hydroxyvitamin D, and offspring body composition: the healthy start study. Nutrients. (2017) 9:790. doi: 10.3390/nu9070790 28737667 PMC5537904

[39] Weiler H Fitzpatrick-Wong S Veitch R Kovacs H Schellenberg J McCloy U. Vitamin D deficiency and whole-body and femur bone mass relative to weight in healthy newborns. Cmaj. (2005) 172:757–61. doi: 10.1503/cmaj.1040508 PMC552889 15767609

[40] Oktaria V Graham SM Triasih R Soenarto Y Bines JE Ponsonby AL. The prevalence and determinants of vitamin D deficiency in Indonesian infants at birth and six months of age. PloS One. (2020) 15:e0239603. doi: 10.1371/journal.pone.0239603 33017838 PMC7535980

[41] Zittermann A Pilz S Berthold HK. Serum 25-hydroxyvitamin D response to vitamin D supplementation in infants: A systematic review and meta-analysis of clinical intervention trials. Eur J Nutr. (2020) 59:359–69. doi: 10.1007/s00394-019-01912-x 30721411

[42] Szymczak-Pajor I Miazek K Selmi A Balcerczyk A Śliwińska A. The action of vitamin D in adipose tissue: is there the link between vitamin D deficiency and adipose tissue-related metabolic disorders? Int J Mol Sci. (2022) 23:956. doi: 10.3390/ijms23020956 35055140 PMC8779075

[43] Ma K Wei SQ Bi WG Weiler HA Wen SW. Effect of vitamin D supplementation in early life on children's growth and body composition: A systematic review and meta-analysis of randomized controlled trials. Nutrients. (2021) 13:524. doi: 10.3390/nu13020524 33562750 PMC7914476

